# Bis(2,2′-bipyrid­yl)(dichloro­acetato)copper(II) dichloro­acetate dihydrate

**DOI:** 10.1107/S1600536809055640

**Published:** 2010-01-13

**Authors:** Yu-Feng Li, Lin-Tong Wang, Fang-Fang Jian

**Affiliations:** a Microscale Science Institute, Department of Chemistry and Chemical Engineering, Weifang University, Weifang 261061, People’s Republic of China; b Department of Chemistry and Chemical Engineering, Weifang University, Weifang 261061, People’s Republic of China; c Microscale Science Institute, Weifang University, Weifang 261061, People’s Republic of China

## Abstract

In the title compound, [Cu(C_2_HCl_2_O_2_)(C_10_H_8_N_2_)_2_](C_2_HCl_2_O_2_)·2H_2_O, the Cu^II^ ion is bonded to two *N*,*N*′-bidentate 2,2′-bipyridyl ligands and one *O*-monodentate 2,2-dichloro­acetate anion in a distorted CuON_4_ trigonal-bipyramidal geometry, with the O atom occupying an equatorial site. In the crystal, the components are linked by O—H⋯O and O—H⋯Cl hydrogen bonds.

## Related literature

For a related structure, see: Barszcz *et al.* (2004 ▶).
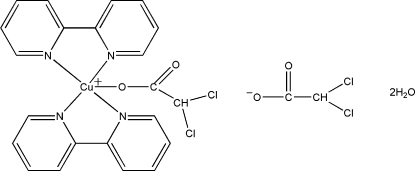


## Experimental

### 

#### Crystal data

[Cu(C_2_HCl_2_O_2_)(C_10_H_8_N_2_)_2_](C_2_HCl_2_O_2_)·2H_2_O
*M* *_r_* = 667.80
Triclinic, 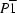

*a* = 9.9710 (7) Å
*b* = 11.7307 (9) Å
*c* = 12.4736 (9) Å
α = 105.407 (1)°
β = 101.499 (1)°
γ = 95.513 (1)°
*V* = 1361.07 (17) Å^3^
*Z* = 2
Mo *K*α radiation
μ = 1.24 mm^−1^
*T* = 293 K
0.22 × 0.20 × 0.18 mm

#### Data collection

Bruker SMART CCD diffractometer
7789 measured reflections
5280 independent reflections
4592 reflections with *I* > 2σ(*I*)
*R*_int_ = 0.033

#### Refinement

*R*[*F* ^2^ > 2σ(*F* ^2^)] = 0.034
*wR*(*F* ^2^) = 0.097
*S* = 1.06
5280 reflections
365 parameters
7 restraints
H atoms treated by a mixture of independent and constrained refinement
Δρ_max_ = 0.65 e Å^−3^
Δρ_min_ = −0.64 e Å^−3^

### 

Data collection: *SMART* (Bruker, 1997 ▶); cell refinement: *SAINT* (Bruker, 1997 ▶); data reduction: *SAINT*; program(s) used to solve structure: *SHELXS97* (Sheldrick, 2008 ▶); program(s) used to refine structure: *SHELXL97* (Sheldrick, 2008 ▶); molecular graphics: *SHELXTL* (Sheldrick, 2008 ▶); software used to prepare material for publication: *SHELXTL*.

## Supplementary Material

Crystal structure: contains datablocks global, I. DOI: 10.1107/S1600536809055640/hb5295sup1.cif

Structure factors: contains datablocks I. DOI: 10.1107/S1600536809055640/hb5295Isup2.hkl

Additional supplementary materials:  crystallographic information; 3D view; checkCIF report

## Figures and Tables

**Table 1:** Selected bond lengths (Å)

Cu1—N1	1.9879 (18)
Cu1—N4	1.9889 (18)
Cu1—O1	2.0121 (16)
Cu1—N3	2.0711 (18)
Cu1—N2	2.1201 (18)

**Table 2:** Hydrogen-bond geometry (Å, °)

*D*—H⋯*A*	*D*—H	H⋯*A*	*D*⋯*A*	*D*—H⋯*A*
O1*W*—H1*A*⋯Cl2	0.84 (2)	2.79 (2)	3.571 (3)	155 (4)
O1*W*—H1*B*⋯O4	0.86 (2)	1.93 (2)	2.787 (4)	171 (4)
O2*W*—H2*A*⋯Cl1	0.88 (2)	2.76 (2)	3.511 (3)	144 (3)
O2*W*—H2*B*⋯O3^i^	0.87 (2)	1.89 (2)	2.757 (3)	172 (4)

Symmetry code: (i) 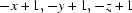.

**Fig. 1.**
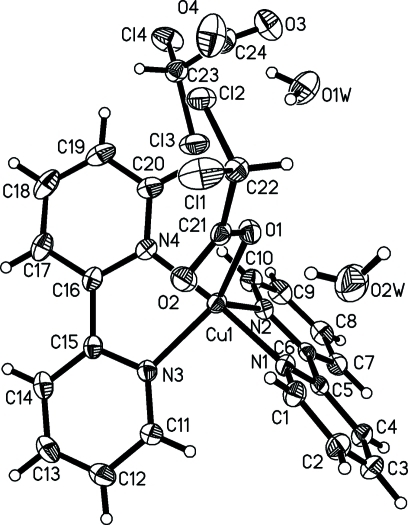
The molecular structure for (I), with displacement ellipsoids drawn at the 30% probability level.

## supplementary crystallographic information

### Experimental

Copper(II) 2,2-dicholoracetate, 0.32 g (1 mmol) and 2,2'-bipyridine 0.31 g (2 mmol) were added to 65 ml anhydrous alcohol under stirring. The mixture was refluxed for 7 h. The blue solution was filtered and the filtrate was left to stand undisturbed. Upon slow evaporation at room temperature, blue blocks of (I) appeared three days later and were separated by filtration.

### Refinement

The water H atoms were located in a difference map and freely refined. The C-bonded H atoms were positioned geometrically (C—H = 0.93–0.98 Å) and refined as riding with *U*_iso_(H) = 1.2*U*_eq_(C).

### Crystal data

**Table:**

[Cu(C_2_HCl_2_O_2_)(C_10_H_8_N_2_)_2_](C_2_HCl_2_O_2_)·2H_2_O	*Z* = 2
*M**_r_* = 667.80	*F*(000) = 678
Triclinic, *P*1	*D*_x_ = 1.629 Mg m^−^^3^
Hall symbol: -P 1	Mo *K*α radiation, λ = 0.71073 Å
*a* = 9.9710 (7) Å	Cell parameters from 2240 reflections
*b* = 11.7307 (9) Å	θ = 2.2–28.3°
*c* = 12.4736 (9) Å	µ = 1.24 mm^−^^1^
α = 105.407 (1)°	*T* = 293 K
β = 101.499 (1)°	Block, blue
γ = 95.513 (1)°	0.22 × 0.20 × 0.18 mm
*V* = 1361.07 (17) Å^3^	

### Data collection

**Table:**

Bruker SMART CCD diffractometer	4592 reflections with *I* > 2σ(*I*)
Radiation source: fine-focus sealed tube	*R*_int_ = 0.033
graphite	θ_max_ = 26.0°, θ_min_ = 1.8°
phi and ω scans	*h* = −11→12
7789 measured reflections	*k* = −13→14
5280 independent reflections	*l* = −15→14

### Refinement

**Table:**

Refinement on *F*^2^	Secondary atom site location: difference Fourier map
Least-squares matrix: full	Hydrogen site location: inferred from neighbouring sites
*R*[*F*^2^ > 2σ(*F*^2^)] = 0.034	H atoms treated by a mixture of independent and constrained refinement
*wR*(*F*^2^) = 0.097	*w* = 1/[σ^2^(*F*_o_^2^) + (0.0542*P*)^2^ + 0.3598*P*] where *P* = (*F*_o_^2^ + 2*F*_c_^2^)/3
*S* = 1.06	(Δ/σ)_max_ < 0.001
5280 reflections	Δρ_max_ = 0.65 e Å^−^^3^
365 parameters	Δρ_min_ = −0.64 e Å^−^^3^
7 restraints	Extinction correction: *SHELXL97* (Sheldrick, 2008), Fc^*^=kFc[1+0.001xFc^2^λ^3^/sin(2θ)]^-1/4^
Primary atom site location: structure-invariant direct methods	Extinction coefficient: 0.0256 (15)

### Special details

**Table:**

Geometry. All e.s.d.'s (except the e.s.d. in the dihedral angle between two l.s. planes) are estimated using the full covariance matrix. The cell e.s.d.'s are taken into account individually in the estimation of e.s.d.'s in distances, angles and torsion angles; correlations between e.s.d.'s in cell parameters are only used when they are defined by crystal symmetry. An approximate (isotropic) treatment of cell e.s.d.'s is used for estimating e.s.d.'s involving l.s. planes.
Refinement. Refinement of *F*^2^ against ALL reflections. The weighted *R*-factor *wR* and goodness of fit *S* are based on *F*^2^, conventional *R*-factors *R* are based on *F*, with *F* set to zero for negative *F*^2^. The threshold expression of *F*^2^ > σ(*F*^2^) is used only for calculating *R*-factors(gt) *etc*. and is not relevant to the choice of reflections for refinement. *R*-factors based on *F*^2^ are statistically about twice as large as those based on *F*, and *R*- factors based on ALL data will be even larger.

### Fractional atomic coordinates and isotropic or equivalent isotropic displacement parameters (Å^2^)

**Table:**

	*x*	*y*	*z*	*U*_iso_*/*U*_eq_	
Cu1	0.02070 (3)	0.28505 (2)	0.24318 (2)	0.03074 (11)	
Cl1	0.45242 (8)	0.39464 (9)	0.06435 (8)	0.0741 (3)	
Cl2	0.50350 (6)	0.30032 (6)	0.25678 (6)	0.04867 (17)	
O1	0.21727 (16)	0.36582 (14)	0.27220 (14)	0.0396 (4)	
O2	0.17501 (17)	0.31459 (16)	0.08196 (15)	0.0459 (4)	
N1	−0.06224 (19)	0.43199 (16)	0.24029 (15)	0.0318 (4)	
N2	−0.05985 (19)	0.31277 (16)	0.39146 (15)	0.0326 (4)	
N3	−0.13923 (19)	0.17107 (16)	0.11654 (15)	0.0328 (4)	
N4	0.08416 (19)	0.12907 (17)	0.24147 (16)	0.0347 (4)	
C1	−0.0573 (3)	0.4885 (2)	0.1606 (2)	0.0406 (5)	
H1	−0.0007	0.4659	0.1103	0.049*	
C2	−0.1329 (3)	0.5790 (2)	0.1503 (2)	0.0465 (6)	
H2	−0.1280	0.6162	0.0936	0.056*	
C3	−0.2157 (3)	0.6134 (2)	0.2255 (2)	0.0467 (6)	
H3	−0.2691	0.6732	0.2193	0.056*	
C4	−0.2185 (2)	0.5581 (2)	0.3104 (2)	0.0415 (5)	
H4	−0.2722	0.5816	0.3629	0.050*	
C5	−0.1403 (2)	0.46721 (18)	0.31641 (18)	0.0303 (4)	
C6	−0.1365 (2)	0.40123 (18)	0.40308 (17)	0.0299 (4)	
C7	−0.2055 (2)	0.4282 (2)	0.4908 (2)	0.0404 (5)	
H6	−0.2572	0.4905	0.4982	0.049*	
C8	−0.1960 (3)	0.3608 (2)	0.5670 (2)	0.0460 (6)	
H8	−0.2415	0.3771	0.6263	0.055*	
C9	−0.1187 (3)	0.2699 (2)	0.5541 (2)	0.0472 (6)	
H9	−0.1114	0.2233	0.6041	0.057*	
C10	−0.0515 (3)	0.2483 (2)	0.4653 (2)	0.0411 (5)	
H10	0.0013	0.1868	0.4569	0.049*	
C11	−0.2514 (2)	0.1998 (2)	0.0568 (2)	0.0405 (5)	
H11	−0.2605	0.2802	0.0702	0.049*	
C12	−0.3538 (3)	0.1155 (3)	−0.0237 (2)	0.0495 (6)	
H12	−0.4300	0.1383	−0.0644	0.059*	
C13	−0.3404 (3)	−0.0027 (3)	−0.0422 (2)	0.0525 (7)	
H13	−0.4082	−0.0614	−0.0960	0.063*	
C14	−0.2268 (3)	−0.0348 (2)	0.0188 (2)	0.0457 (6)	
H14	−0.2175	−0.1150	0.0072	0.055*	
C15	−0.1260 (2)	0.05451 (19)	0.09785 (18)	0.0334 (5)	
C16	0.0007 (2)	0.0309 (2)	0.16703 (19)	0.0351 (5)	
C17	0.0356 (3)	−0.0829 (2)	0.1571 (2)	0.0478 (6)	
H17	−0.0222	−0.1500	0.1051	0.057*	
C18	0.1576 (3)	−0.0946 (3)	0.2256 (3)	0.0571 (8)	
H18	0.1831	−0.1698	0.2199	0.069*	
C19	0.2412 (3)	0.0060 (3)	0.3024 (2)	0.0528 (7)	
H19	0.3232	−0.0005	0.3495	0.063*	
C20	0.2015 (3)	0.1165 (2)	0.3086 (2)	0.0450 (6)	
H20	0.2577	0.1843	0.3607	0.054*	
C21	0.2517 (2)	0.35388 (19)	0.1777 (2)	0.0341 (5)	
C22	0.4076 (2)	0.3961 (2)	0.1938 (2)	0.0402 (5)	
H22	0.4324	0.4778	0.2456	0.048*	
Cl3	0.17481 (9)	0.06640 (8)	0.59492 (10)	0.0833 (3)	
Cl4	0.36379 (9)	−0.04850 (8)	0.72163 (7)	0.0722 (2)	
O3	0.4801 (3)	0.2075 (2)	0.7501 (2)	0.0807 (7)	
O4	0.4943 (3)	0.1598 (2)	0.5687 (3)	0.0971 (9)	
C23	0.3436 (3)	0.0293 (2)	0.6185 (2)	0.0466 (6)	
H23A	0.3545	−0.0240	0.5466	0.056*	
C24	0.4501 (3)	0.1442 (3)	0.6506 (3)	0.0550 (7)	
O1W	0.4574 (3)	0.3842 (2)	0.5424 (2)	0.0744 (7)	
H1A	0.455 (4)	0.384 (4)	0.4747 (19)	0.089*	
H1B	0.467 (4)	0.312 (2)	0.543 (3)	0.089*	
O2W	0.2759 (3)	0.6333 (2)	0.1579 (3)	0.0885 (8)	
H2A	0.299 (3)	0.579 (2)	0.104 (2)	0.106*	
H2B	0.349 (3)	0.686 (3)	0.192 (3)	0.106*	

### Atomic displacement parameters (Å^2^)

**Table:**

	*U*^11^	*U*^22^	*U*^33^	*U*^12^	*U*^13^	*U*^23^
Cu1	0.03054 (16)	0.02735 (16)	0.03555 (17)	0.00635 (10)	0.00873 (11)	0.00987 (11)
Cl1	0.0572 (4)	0.1167 (7)	0.0791 (5)	0.0245 (5)	0.0366 (4)	0.0615 (5)
Cl2	0.0370 (3)	0.0532 (4)	0.0565 (4)	0.0097 (3)	0.0046 (3)	0.0210 (3)
O1	0.0362 (8)	0.0390 (9)	0.0408 (9)	0.0010 (7)	0.0126 (7)	0.0062 (7)
O2	0.0399 (9)	0.0490 (10)	0.0437 (10)	0.0044 (8)	0.0057 (8)	0.0091 (8)
N1	0.0333 (9)	0.0273 (9)	0.0355 (9)	0.0062 (7)	0.0103 (8)	0.0083 (7)
N2	0.0341 (9)	0.0306 (9)	0.0314 (9)	0.0031 (7)	0.0070 (8)	0.0073 (7)
N3	0.0332 (9)	0.0300 (9)	0.0337 (9)	0.0034 (7)	0.0085 (8)	0.0070 (7)
N4	0.0354 (10)	0.0352 (10)	0.0375 (10)	0.0100 (8)	0.0118 (8)	0.0130 (8)
C1	0.0478 (14)	0.0352 (12)	0.0423 (13)	0.0085 (10)	0.0143 (11)	0.0140 (10)
C2	0.0562 (16)	0.0365 (13)	0.0501 (14)	0.0084 (11)	0.0097 (12)	0.0199 (11)
C3	0.0469 (14)	0.0349 (13)	0.0582 (16)	0.0147 (11)	0.0073 (12)	0.0140 (11)
C4	0.0384 (12)	0.0368 (13)	0.0480 (14)	0.0108 (10)	0.0112 (11)	0.0076 (10)
C5	0.0250 (10)	0.0268 (10)	0.0339 (11)	−0.0008 (8)	0.0038 (8)	0.0040 (8)
C6	0.0251 (10)	0.0287 (10)	0.0300 (10)	−0.0027 (8)	0.0036 (8)	0.0031 (8)
C7	0.0343 (12)	0.0429 (13)	0.0396 (12)	0.0027 (10)	0.0116 (10)	0.0034 (10)
C8	0.0433 (14)	0.0579 (16)	0.0347 (12)	−0.0009 (12)	0.0162 (11)	0.0078 (11)
C9	0.0540 (15)	0.0526 (15)	0.0374 (13)	−0.0002 (12)	0.0121 (11)	0.0191 (11)
C10	0.0464 (14)	0.0402 (13)	0.0401 (13)	0.0074 (10)	0.0115 (11)	0.0160 (10)
C11	0.0361 (12)	0.0399 (13)	0.0423 (13)	0.0057 (10)	0.0047 (10)	0.0100 (10)
C12	0.0372 (13)	0.0603 (17)	0.0445 (14)	0.0023 (12)	0.0023 (11)	0.0119 (12)
C13	0.0465 (15)	0.0520 (16)	0.0445 (14)	−0.0091 (12)	0.0039 (12)	0.0008 (12)
C14	0.0526 (15)	0.0332 (12)	0.0450 (14)	−0.0018 (11)	0.0142 (12)	0.0018 (10)
C15	0.0377 (12)	0.0306 (11)	0.0328 (11)	0.0034 (9)	0.0149 (9)	0.0068 (9)
C16	0.0426 (12)	0.0314 (11)	0.0382 (12)	0.0084 (10)	0.0209 (10)	0.0123 (9)
C17	0.0645 (17)	0.0339 (13)	0.0553 (15)	0.0157 (12)	0.0273 (13)	0.0179 (11)
C18	0.081 (2)	0.0492 (16)	0.0656 (18)	0.0368 (15)	0.0391 (17)	0.0323 (14)
C19	0.0521 (16)	0.0683 (19)	0.0564 (16)	0.0319 (14)	0.0212 (13)	0.0349 (15)
C20	0.0412 (13)	0.0545 (15)	0.0449 (13)	0.0157 (12)	0.0108 (11)	0.0207 (12)
C21	0.0345 (11)	0.0244 (10)	0.0450 (13)	0.0052 (9)	0.0116 (10)	0.0110 (9)
C22	0.0361 (12)	0.0368 (12)	0.0500 (14)	0.0044 (10)	0.0134 (11)	0.0148 (11)
Cl3	0.0527 (4)	0.0712 (5)	0.1169 (8)	0.0183 (4)	−0.0088 (5)	0.0304 (5)
Cl4	0.0675 (5)	0.0819 (6)	0.0722 (5)	0.0014 (4)	−0.0030 (4)	0.0485 (4)
O3	0.0808 (17)	0.0708 (16)	0.0752 (16)	−0.0105 (13)	0.0049 (13)	0.0133 (13)
O4	0.139 (3)	0.0615 (15)	0.108 (2)	0.0046 (15)	0.068 (2)	0.0287 (14)
C23	0.0518 (15)	0.0491 (15)	0.0428 (13)	0.0156 (12)	0.0092 (12)	0.0187 (11)
C24	0.0577 (17)	0.0466 (16)	0.0661 (19)	0.0142 (13)	0.0198 (15)	0.0196 (14)
O1W	0.0842 (16)	0.0731 (16)	0.0853 (17)	0.0306 (13)	0.0462 (15)	0.0298 (14)
O2W	0.0756 (17)	0.0565 (15)	0.130 (2)	0.0066 (12)	0.0219 (17)	0.0243 (15)

### Geometric parameters (Å, °)

**Table:**

Cu1—N1	1.9879 (18)	C9—H9	0.9300
Cu1—N4	1.9889 (18)	C10—H10	0.9300
Cu1—O1	2.0121 (16)	C11—C12	1.378 (3)
Cu1—N3	2.0711 (18)	C11—H11	0.9300
Cu1—N2	2.1201 (18)	C12—C13	1.368 (4)
Cl1—C22	1.755 (3)	C12—H12	0.9300
Cl2—C22	1.780 (2)	C13—C14	1.377 (4)
O1—C21	1.268 (3)	C13—H13	0.9300
O2—C21	1.225 (3)	C14—C15	1.390 (3)
N1—C1	1.340 (3)	C14—H14	0.9300
N1—C5	1.352 (3)	C15—C16	1.476 (3)
N2—C10	1.333 (3)	C16—C17	1.391 (3)
N2—C6	1.341 (3)	C17—C18	1.382 (4)
N3—C11	1.339 (3)	C17—H17	0.9300
N3—C15	1.349 (3)	C18—C19	1.377 (4)
N4—C20	1.341 (3)	C18—H18	0.9300
N4—C16	1.350 (3)	C19—C20	1.379 (4)
C1—C2	1.378 (3)	C19—H19	0.9300
C1—H1	0.9300	C20—H20	0.9300
C2—C3	1.377 (4)	C21—C22	1.542 (3)
C2—H2	0.9300	C22—H22	0.9800
C3—C4	1.385 (4)	Cl3—C23	1.766 (3)
C3—H3	0.9300	Cl4—C23	1.757 (3)
C4—C5	1.388 (3)	O3—C24	1.225 (4)
C4—H4	0.9300	O4—C24	1.239 (4)
C5—C6	1.484 (3)	C23—C24	1.540 (4)
C6—C7	1.388 (3)	C23—H23A	0.9800
C7—C8	1.384 (4)	O1W—H1A	0.839 (18)
C7—H6	0.9300	O1W—H1B	0.861 (19)
C8—C9	1.369 (4)	O2W—H2A	0.884 (16)
C8—H8	0.9300	O2W—H2B	0.867 (18)
C9—C10	1.385 (3)		
			
N1—Cu1—N4	174.19 (8)	C9—C10—H10	118.8
N1—Cu1—O1	94.77 (7)	N3—C11—C12	122.9 (2)
N4—Cu1—O1	91.01 (7)	N3—C11—H11	118.5
N1—Cu1—N3	94.63 (7)	C12—C11—H11	118.5
N4—Cu1—N3	80.37 (7)	C13—C12—C11	118.3 (3)
O1—Cu1—N3	142.56 (7)	C13—C12—H12	120.9
N1—Cu1—N2	79.44 (7)	C11—C12—H12	120.9
N4—Cu1—N2	98.73 (7)	C12—C13—C14	120.0 (2)
O1—Cu1—N2	114.17 (7)	C12—C13—H13	120.0
N3—Cu1—N2	103.17 (7)	C14—C13—H13	120.0
C21—O1—Cu1	109.56 (14)	C13—C14—C15	119.0 (2)
C1—N1—C5	119.18 (19)	C13—C14—H14	120.5
C1—N1—Cu1	123.79 (15)	C15—C14—H14	120.5
C5—N1—Cu1	116.62 (15)	N3—C15—C14	121.1 (2)
C10—N2—C6	118.93 (19)	N3—C15—C16	115.17 (19)
C10—N2—Cu1	127.95 (16)	C14—C15—C16	123.8 (2)
C6—N2—Cu1	112.90 (14)	N4—C16—C17	121.1 (2)
C11—N3—C15	118.69 (19)	N4—C16—C15	115.04 (19)
C11—N3—Cu1	127.97 (16)	C17—C16—C15	123.8 (2)
C15—N3—Cu1	113.30 (14)	C18—C17—C16	119.0 (3)
C20—N4—C16	119.4 (2)	C18—C17—H17	120.5
C20—N4—Cu1	124.53 (17)	C16—C17—H17	120.5
C16—N4—Cu1	116.08 (15)	C19—C18—C17	119.5 (2)
N1—C1—C2	122.5 (2)	C19—C18—H18	120.3
N1—C1—H1	118.7	C17—C18—H18	120.3
C2—C1—H1	118.7	C18—C19—C20	119.1 (3)
C3—C2—C1	118.8 (2)	C18—C19—H19	120.5
C3—C2—H2	120.6	C20—C19—H19	120.5
C1—C2—H2	120.6	N4—C20—C19	121.9 (3)
C2—C3—C4	119.2 (2)	N4—C20—H20	119.0
C2—C3—H3	120.4	C19—C20—H20	119.0
C4—C3—H3	120.4	O2—C21—O1	126.7 (2)
C3—C4—C5	119.4 (2)	O2—C21—C22	120.8 (2)
C3—C4—H4	120.3	O1—C21—C22	112.5 (2)
C5—C4—H4	120.3	C21—C22—Cl1	112.61 (17)
N1—C5—C4	120.8 (2)	C21—C22—Cl2	108.80 (16)
N1—C5—C6	115.24 (18)	Cl1—C22—Cl2	108.94 (13)
C4—C5—C6	123.9 (2)	C21—C22—H22	108.8
N2—C6—C7	121.7 (2)	Cl1—C22—H22	108.8
N2—C6—C5	114.97 (18)	Cl2—C22—H22	108.8
C7—C6—C5	123.4 (2)	C24—C23—Cl4	113.67 (19)
C8—C7—C6	118.9 (2)	C24—C23—Cl3	109.08 (18)
C8—C7—H6	120.6	Cl4—C23—Cl3	109.69 (15)
C6—C7—H6	120.6	C24—C23—H23A	108.1
C9—C8—C7	119.2 (2)	Cl4—C23—H23A	108.1
C9—C8—H8	120.4	Cl3—C23—H23A	108.1
C7—C8—H8	120.4	O3—C24—O4	128.5 (3)
C8—C9—C10	119.0 (2)	O3—C24—C23	118.1 (3)
C8—C9—H9	120.5	O4—C24—C23	113.4 (3)
C10—C9—H9	120.5	H1A—O1W—H1B	104 (4)
N2—C10—C9	122.3 (2)	H2A—O2W—H2B	107 (2)
N2—C10—H10	118.8		

### Hydrogen-bond geometry (Å, °)

**Table:**

*D*—H···*A*	*D*—H	H···*A*	*D*···*A*	*D*—H···*A*
O1W—H1A···Cl2	0.84 (2)	2.79 (2)	3.571 (3)	155 (4)
O1W—H1B···O4	0.86 (2)	1.93 (2)	2.787 (4)	171 (4)
O2W—H2A···Cl1	0.88 (2)	2.76 (2)	3.511 (3)	144 (3)
O2W—H2B···O3^i^	0.87 (2)	1.89 (2)	2.757 (3)	172 (4)

Symmetry codes: (i) −*x*+1, −*y*+1, −*z*+1.
